# Bilateral retrovesical splenosis following childhood splenectomy mimicking pelvic malignancy: A video case report

**DOI:** 10.1016/j.eucr.2026.103502

**Published:** 2026-06-06

**Authors:** Amir Naiem, Raphael Röthlisberger, Nicolas Arnold, Livio Mordasini, Laila Schneidewind, Beat Roth, Nicola Giudici

**Affiliations:** a Inselspital, Department of Urology, Freiburgstrasse 37, 3010, Bern, Switzerland; b Uroviva AG, Urologie-Praxis Sursee, ChristophSchnyder-Strasse 2b, 6210, Sursee, Switzerland

## Abstract

Splenosis is a benign condition resulting from heterotopic autotransplantation of splenic tissue after splenic rupture or splenectomy, most commonly following trauma. Although usually asymptomatic, it may mimic malignancy on imaging, particularly in uncommon anatomical locations. We report a 31-year-old male presenting with intermittent terminal macroscopic hematuria. Imaging revealed two bilateral retrovesical masses posterior to the seminal vesicles. Given diagnostic uncertainty, robot-assisted excision was performed. Histopathology confirmed bilateral retrovesical splenosis. This case highlights retrovesical splenosis as a rare diagnostic pitfall in patients with prior splenectomy.

## Highlights

• Bilateral retrovesical splenosis mimicking pelvic malignancy.
• Occurred decades after childhood splenectomy.
• Presented with intermittent terminal macroscopic hematuria.
• Cross-sectional imaging could not exclude malignancy.
• Robot-assisted excision enabled definitive diagnosis with organ preservation.

## 1 Introduction

Splenosis is defined as the heterotopic autotransplantation of splenic tissue following splenic rupture or splenectomy, most commonly after traumatic injury.^1^^,^^2^ Viable splenic tissue may implant at distant sites and persist for decades, usually without causing symptoms. The condition is therefore often discovered incidentally during imaging or surgery performed for unrelated reasons.^2^

The clinical relevance of splenosis lies in its ability to mimic malignant or other pathological processes on cross-sectional imaging, potentially leading to extensive diagnostic workup or unnecessary surgical intervention.^2^ ^3^ Pelvic splenosis is uncommon, and retrovesical localisation is particularly rare, with only isolated cases reported in the literature.^3^ ^4^

In male patients, retrovesical or paraprostatic masses raise a broad differential diagnosis, including ectopic testicular tissue, Müllerian duct remnants, paraganglioma, lymphadenopathy, and other benign or malignant pelvic tumours.^4^ Awareness of splenosis as a differential diagnosis is essential, especially in patients with a history of splenic trauma or splenectomy; however, despite this awareness, diagnostic uncertainty often persists, and surgical excision is frequently required to establish a definitive diagnosis.

We report a rare case of bilateral retrovesical splenosis presenting with intermittent terminal macroscopic hematuria, ultimately diagnosed after robot-assisted surgical excision.

## 2 Case report

A 31-year-old non-smoking male presented in January 2025 with intermittent terminal macroscopic hematuria, first noted approximately 18 months earlier, with recurrent episodes three months and one week prior to presentation. He denied lower urinary tract symptoms, genital trauma, or previous urological investigations. His medical history was unremarkable except for an emergency splenectomy performed in childhood following abdominal trauma.

Physical examination revealed no abnormal findings. Ultrasound demonstrated normal kidneys without pelvicalyceal dilatation and no indication of residual urine or tumor in the bladder. Flexible cystoscopy revealed a non-obstructive bilobular prostate without visible intravesical lesions, while bladder wash cytology showed atypical cells. To further investigate the cytological findings and to evaluate the upper urinary tract, a three-phase CT urography was performed in February 2025. It revealed two well-circumscribed oval soft-tissue masses measuring approximately 2.7–2.8 cm in the rectovesical pouch, located posterior and cranial to the seminal vesicles (Fig. 1.). No additional pathological findings were identified based on these results, further evaluation with pelvic MRI was performed (Fig. 2. ABC). MRI confirmed two sharply demarcated, homogeneous soft-tissue lesions paramedian and bilateral, positioned posterior and cranial to the prostate and the seminal vesicles, without evidence of surrounding inflammatory changes or lymphadenopathy. Differential diagnoses included ectopic testicular tissue, Müllerian duct remnants, paraganglioma, or other benign idiopathic lesions, with no radiological signs of malignancy.

**Fig. 1:**
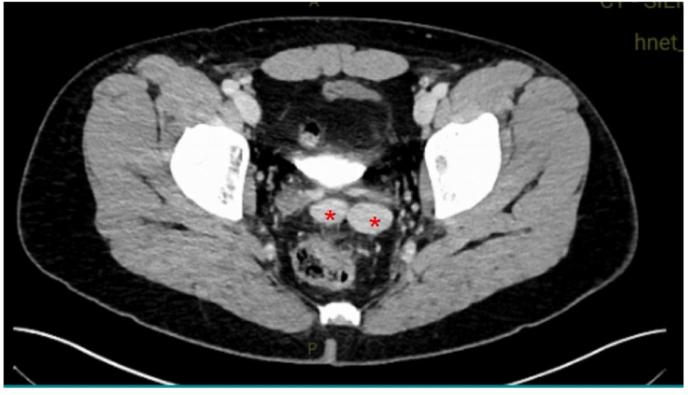
Axial contrast-enhanced CT image demonstrating two well-circumscribed oval soft-tissue masses in the rectovesical ponch located posterior and cranial to the seminal vesicles.

**Fig. 2:**
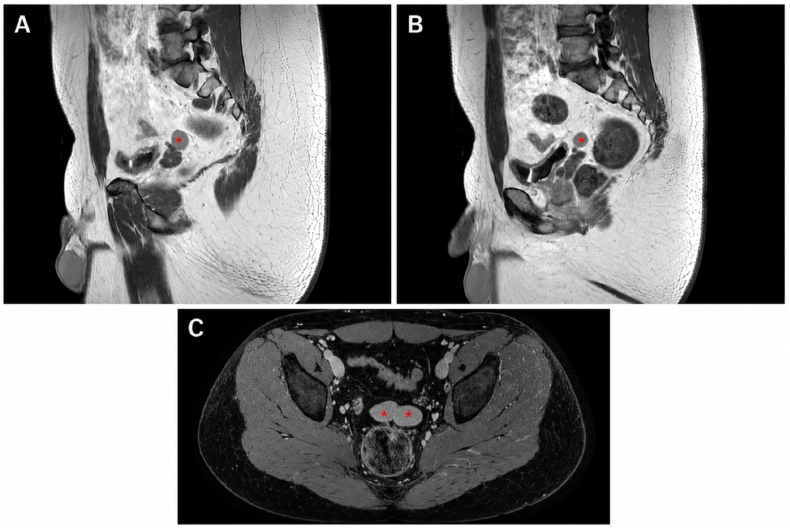
Presecral space-occupying lesion on MRI (A) Saggittal T2-weighted image:Presecral space-occupying lesion (red star) (b) Saggittal T2-weighted image (adjacent slice):Presecral space-occupying lesion (red star) (c) Axial T2-weighted fat saturated image: Presecral space-occupying lesion (red stars).

In March 2025, the patient was referred to our university hospital for second opinion and diagnostic workup. Rigid cystoscopy and bladder biopsies were negative for malignancy. Given the imaging appearance and anatomical location of the lesions, paraganglioma was considered; however, the patient was asymptomatic and normotensive, with no catecholamine-related symptoms. The decision for robot-assisted surgical excision to obtain a definitive diagnosis was made; however, surgical intervention was deferred due to the patient's desire for conception and was postponed until December 2025 after successful pregnancy of his partner.

### 2.1 Surgical technique (Video 1.)

The patient was positioned in supine Trendelenburg position. After standard skin preparation and draping, perioperative antibiotic prophylaxis with intravenous cefuroxime was administered. Access to the abdominal cavity was obtained through a supraumbilical mini-laparotomy, followed by placement of the camera trocar and establishment of pneumoperitoneum. Four robotic working ports were then placed in a standard fan-shaped configuration across the lower abdomen, ensuring adequate spacing for optimal instrument articulation. Two additional assistant ports were positioned laterally and cranially on the left side to allow suction and retraction (Fig. 3).

**Fig. 3:**
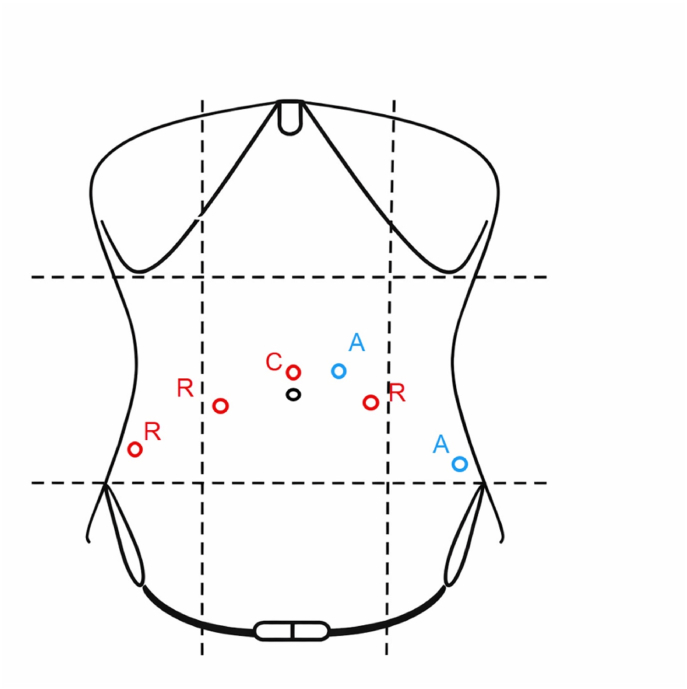
Port placement: A:Assistant port; C:Camera; R:Robotic port.

Initial laparoscopic exploration revealed intra-abdominal adhesions consistent with the patient's prior splenectomy. After docking of the robotic system, the peritoneum was incised to access the retrovesical space. Two well-circumscribed, dark-red nodular lesions were identified posterior to the seminal vesicles, corresponding to the preoperative imaging findings. The lesions were carefully dissected using a combination of sharp and blunt techniques. Particular attention was paid to preserve the seminal vesicles and surrounding structures, which were not injured during the procedure. Hemostasis was achieved, and both specimens were removed intact. The peritoneal defect was subsequently closed using a running 3-0 Vicryl suture. The operative field was irrigated, and the trocars were removed under direct vision. The specimens were retrieved, and the abdominal wall was closed in layers, with skin closure performed using skin glue.

The postoperative course was uneventful. The indwelling urinary catheter was removed on postoperative day one, and the patient was discharged the same day. Histopathological examination confirmed splenic tissue consistent with bilateral retrovesical splenosis

## 3 Discussion

We present an unusual case of splenosis manifesting as bilateral retrovesical masses in a young male patient with a history of childhood splenectomy. The lesions were incidentally detected during the evaluation of intermittent terminal macroscopic hematuria. Due to their imaging characteristics, size, and shape, invasive diagnostic excision was performed to determine their nature.

Splenosis is generally asymptomatic and most often identified incidentally years or decades after splenic injury.^1^^,^^2^ Several reports have described retrovesical splenosis initially misinterpreted as ectopic testicular tissue or pelvic malignancy, with the correct diagnosis established only after histological examination.^3^ ^4^ When symptoms occur, they are usually related to mass effect or bleeding caused by the presence of ectopic splenic tissue.^5^ Interestingly, in the present case, a direct causal relationship between splenosis and hematuria could not be definitively established, as no direct involvement of the urinary tract was identified. However, we hypothesize that both the macroscopic hematuria and the presence of atypical cells on urinary cytology may have resulted from connection between the splenic tissue and the seminal vesicles, potentially explaining both findings. Notably, endoscopic evaluation did not reveal any pathological lesions within the bladder. Urinary symptoms, including hematuria, have previously been reported in association with pelvic splenosis and may prompt extensive urological investigation.^5^

While non-invasive nuclear medicine techniques can reliably identify splenic tissue, they are not routinely employed when lesions are located in unusual anatomical regions or when malignancy cannot be confidently excluded.^6^ ^2^ In such cases, surgical excision remains justified. Robot-assisted surgery offers excellent visualisation of the retrovesical space and allows precise dissection with preservation of adjacent structures, as demonstrated in this case and in previous reports.^7^ ^8^

This case highlights the importance of considering splenosis in the differential diagnosis of unexplained pelvic masses in patients with a history of splenic trauma or splenectomy, regardless of the time elapsed since the initial event. Increased awareness of this entity may help clinicians interpret atypical imaging findings more cautiously and avoid misclassification as malignant disease. Nevertheless, despite growing awareness and the availability of advanced imaging modalities, surgical excision often cannot be omitted, particularly in young, otherwise healthy patients in whom diagnostic certainty is required and long-term oncological implications must be excluded. In contrast, in carefully selected older or frail patients with a clear history of splenic trauma, stable imaging findings, and no concerning clinical features, a watchful waiting approach with close radiological follow-up may be considered when the risks of surgical intervention outweigh its potential benefits.

## 4 Conclusion

Retrovesical splenosis is a rare and diagnostically challenging condition that can closely mimic malignant pelvic masses. In patients with a history of splenic trauma or splenectomy, splenosis should be included in the differential diagnosis of unexplained pelvic soft-tissue lesions, even decades after the initial event. When non-invasive diagnostic confirmation is not feasible, robot-assisted surgical excision represents a safe and effective strategy to achieve definitive diagnosis while preserving adjacent pelvic structures.

## CRediT authorship contribution statement

**Amir Naiem:** Writing – original draft, Conceptualization, Project administration, Writing – review & editing, Formal analysis. **Raphael Röthlisberger:** Writing – review & editing. **Nicolas Arnold:** Writing – review & editing. **Livio Mordasini:** Writing – review & editing. **Laila Schneidewind:** Writing – review & editing. **Beat Roth:** Writing – review & editing. **Nicola Giudici:** Conceptualization, Writing – original draft, Writing – review & editing, Formal analysis, Project administration.

## Ethics approval and consent to participate

The patient gave full consent for publication, and written informed consent was obtained through the general consent of Inselspital, Bern University Hospital, which includes permission for the use of anonymized health-related data in scientific publications.

## Consent for publication

Consent was obtained from the patient for publication.

## Availability of data and materials

The data, figures and medical records are included in this case report. Additional details of the remaining data used during the current study are available from the corresponding author upon reasonable request.

## Declaration of generative AI use

During the preparation of this manuscript, generative AI was used solely for English language translation and grammatical editing. The authors reviewed and edited the content as needed and take full responsibility for the content of the published article.

## Clinical trial number

Not applicable.

## Funding

This research did not receive any specific grant from funding agencies in the public, commercial, or not-for-profit sectors.

## Competing interests

The authors declare that they have no competing interests.
